# Women’s intention to screen and willingness to vaccinate their daughters against cervical cancer – a cross sectional study in eastern Uganda

**DOI:** 10.1186/s12889-017-4180-4

**Published:** 2017-03-14

**Authors:** Rawlance Ndejjo, Trasias Mukama, Geofrey Musinguzi, Abdullah Ali Halage, John C. Ssempebwa, David Musoke

**Affiliations:** Department of Disease Control and Environmental Health, School of Public Health, College of Health Sciences, Makerere University, Kampala, Uganda

**Keywords:** Cervical cancer, Intention to screen, Rural, Screening, Uganda, Vaccination, Willingness

## Abstract

**Background:**

The World Health Organization recommends cervical cancer screening and vaccination programmes as measures to combat cervical cancer. The uptake of these measures remains low in Uganda, most especially in rural areas. An understanding of the factors that influence women’s decision to attend screening, and willingness to have their daughters vaccinated against cervical cancer is essential for any attempts to increase uptake of these services. This study assessed the factors associated with intention to screen for cervical cancer among women in eastern Uganda, and willingness to have their daughters vaccinated against the disease.

**Methods:**

This cross sectional study involved 900 females aged 25 to 49 years in Bugiri and Mayuge districts in eastern Uganda. Data were collected using a pretested semi-structured questionnaire, entered in Epidata version 3.02 and analysed in STATA version 12.0. Unadjusted and adjusted prevalence ratios (PR) were computed using a generalized linear model with Poisson family, and a log link with robust standard errors.

**Results:**

Majority 819 (91.0%) of respondents stated that they intended to go for cervical cancer screening in the subsequent six months. Among them, 603 (73.6%) wanted to know their status, 256 (31.3%) thought it was important, 202 (24.7%) wanted to reduce their chances of getting the disease, and 20 (2.4%) had been told to do so by a health worker. Majority 813 (90.4%) of respondents were willing to vaccinate their daughters against cervical cancer. Higher income (adjusted PR = 1.11, 95% CI: 1.03–1.20), cervical cancer screening status (adjusted PR = 0.81, 95% CI: 0.67–0.99) and knowledge of at least one test for cervical cancer (adjusted PR = 0.92, 95% CI: 0.85–0.98) were significantly associated with intention to screen for cervical cancer. No socio-demographic characteristic was associated with willingness to vaccinate daughters among women.

**Conclusion:**

There is a very high intention to screen and willingness to vaccinate daughters against cervical cancer among women in eastern Uganda. To take advantage of this, there is need to avail opportunities for women to access cervical cancer screening and vaccinations particularly among rural communities.

## Background

Cervical cancer remains a major cause of morbidity and mortality among women in resource constrained settings due to low access to cancer screening and vaccination [1, 2]. Indeed, coverage of cancer screening services is lowest in low and middle income countries [3]. These countries have only a few trained and skilled health workers to carry out screening, and lack healthcare resources to sustain such programmes [4]. Consequently, women with cervical cancer are not identified until they are at an advanced stage of disease, resulting in high morbidity and mortality [4]. On the other hand, many developing countries are still in the initial stages of rolling out cervical cancer vaccination among their populations. For example, in Uganda, cervical cancer vaccinations among girls aged between 9 to 13 years in schools and the community were piloted in 2008 and a national strategic plan launched in 2010 [5, 6]. Other sub-Saharan African countries such as Ghana and Kenya have also rolled out cervical cancer vaccinations among their population [7, 8].

Cervical cancer is potentially preventable, and effective screening programmes can lead to a significant reduction in the morbidity and mortality associated with this cancer [2, 9] especially if detected in early stages [10]. The World Health Organization (WHO) recommends screening and vaccination programmes globally [11]. However, only a small percentage (5–27%) of women in sub-Saharan Africa report having received cervical cancer screening [12–19]. This is even lower in the East African region where the prevalence of human papilloma virus (HPV) (20%) is high, and the incidence rates of cervical cancer are highest [20]. Understanding factors that influence intention to screen for cervical cancer among women is important to inform programmes aimed at increasing utilisation of screening services in sub-Saharan Africa. Previous studies show that women’s intention to screen, and attendance of cervical cancer screening is influenced by: demographic factors such as occupation, living with a partner; attitudinal factors such as perception of risk and disease; social factors for example discussions by health workers; and health system factors such as distance to health facilities and awareness of the service [18, 19].

HPV, which is mainly sexually transmitted, causes most of the cervical cancer cases [21]. Highly effective prophylactic vaccines against HPV −16 and HPV −18 have been shown to prevent over 70% of these cases worldwide [22]. HPV vaccinations can therefore significantly reduce the incidence of cervical cancer among women. Moreover, WHO recommends offering HPV vaccine to girls between 9 and 13 years, prior to sexual debut, as the vaccine is most efficacious if girls have not already been exposed to the virus [23]. The success of the vaccine would depend on levels of acceptability and uptake, which heavily relies on parent’s willingness to have their eligible daughters vaccinated [24]. Prior research among parents has reported predictors of vaccinations of girls to include perception of the severity of the disease, belief of daughter being at risk, higher education and income, attitudes towards vaccines, previous awareness of the vaccine, and recommendations by vaccine providers [7, 25–28]. Barriers to cervical cancer vaccination such as concerns about vaccine safety, danger to daughter, sexual behaviours, and lack of provider recommendation have also been highlighted [7, 25–29]. However, there has been a paucity of such studies in Uganda to provide context specific barriers and inform on-going vaccine promotion campaigns. This study assessed the factors associated with intention to screen for cervical cancer among women in eastern Uganda, and willingness to have their daughters vaccinated against the disease.

## Methods

### Study area

The study was conducted in Bugiri and Mayuge districts in eastern Uganda approximately 150 kilometres from Kampala, the country’s capital city. These predominantly rural districts have a combined area of 10,372 km^2^ and a total population of 856,152 people of whom 51.4% are females [30]. Bugiri district is composed of 9 sub-counties while Mayuge has 7 sub-counties. Majority of people in the districts reside in roofed mud and wattle houses. Residents majorly participate in agriculture for subsistence though a few operate small businesses in trading centres, and others engage in fishing along the shores of Lake Victoria. In the two districts, cervical cancer screening is provided by Bugiri district hospital, a government health facility that also serves neighbouring districts. The hospital provides intermittent screening services and treatment of those diagnosed with the disease. Three (3) private health facilities, 2 in Bugiri and 1 in Mayuge district, also provide cervical cancer screening.

### Study design and population

This was a cross sectional study that employed quantitative data collection methods. The study involved females aged 25 to 49 years in Bugiri and Mayuge districts who had lived in the area for at least six months. A minimum sample size of 845 was determined using the sample size estimation formula for cross sectional studies [31] with Z = 1.96, *p* = 0.5 (used to obtain the maximum sample size since proportion was not known), and a precision of 5% taking into account a design effect of 2.0, and a non-response rate of 10%. The sampling units were households, and only one participant was interviewed per selected household with priority given to household heads or their spouses.

### Sampling

Multi-stage sampling technique was used as follows: five sub-counties were randomly selected from each district. Five villages were then selected from each sub-county using simple random sampling to obtain 25 study villages in each district. Lists of sub-counties and villages were obtained from district officials while village Local Council leaders provided estimates of numbers of households in their villages. The study households were selected using systematic random sampling where the interval for selection was determined by dividing the approximate number of households in a given village by the required number of respondents.

### Data collection

Data were collected using a pretested semi-structured questionnaire that was developed in English, and then translated to *Lusoga,* the main local language of the area. The questionnaire had questions on knowledge and attitudes towards cervical cancer and screening, access to cervical cancer screening and intention to screen, respondent willingness to vaccinate their daughters, and socio-demographic factors whose details have been described elsewhere [19]. The questionnaire was administered by a team of trained Research Assistants at respondents’ homes.

### Data analysis

Data were entered in Epidata version 3.02 (EpiData Association, Denmark) and analysed in STATA version 12.0 (Stata Corp, Texas, USA) statistical software. The outcome variables were intention to screen for cervical cancer in the following six months, and willingness for vaccination of daughters against cervical cancer. Intention to screen was assessed using a question which asked respondents “Do you intend to go for cervical cancer screening in the next six months?” with three responses of ‘Yes’ ‘No’ and “Undecided”. ‘Yes’ and ‘No’ responses formed the binary outcome variable and were assigned 1 and 0 respectively during further analysis. For willingness to vaccinate daughters against cervical cancer, respondents answered to the statement “I would allow my children to be vaccinated against cervical cancer” with response based on a 5 point - Likert scale ranging from strongly disagree to strongly agree. Respondents who gave ‘Strongly agree’ and ‘Agree’ responses were coded as ‘willing to allow their daughters receive vaccination’ and assigned 1 while those who responded with ‘Strongly disagree’ and ‘Disagree’ responses were coded as not willing to allow their daughters to be vaccinated’ (assigned 0). Those who were not sure were dropped in creating this outcome variable. Prevalence ratios (PR) computed using a generalized linear model with Poisson family, and a log link with robust standard errors while applying a backward elimination method were used to measure the association between the outcome and independent variables. PRs were used because the outcomes were highly prevalent (>10%) as odds ratios tend to overestimate the relative risk in such instances [32–34]. Simple models were run to obtain the unadjusted PRs, and then basing on biological plausibility and *p* < 0.05, variables from these models were included in the multivariable model. We present both the unadjusted and adjusted PRs, and their corresponding 95% confidence intervals (CI) as well as p-values. A p-value of less than 0.05 was considered for a statistically significant association.

## Results

### Socio-demographic characteristics of respondents

All the 900 respondents selected took part in the study. Majority of these were from rural areas 610 (67.8%) and married 767 (85.2%). Only a quarter 228 (25.3%) of respondents had attained post primary education (Table 1).

**Table 1:** Socio-demographic characteristics of respondents (*N* = 900)

Characteristic	Categories	n (%)
District	Bugiri	452 (50.2)
	Mayuge	448 (49.8)
Residence	Rural	610 (67.8)
	Semi-urban/urban	290 (32.2)
Age (years)	Mean (SD)	32.9 (±6.7)
	25–39	703 (78.1)
	40–49	197 (21.9)
Education level	None/primary	672 (74.7)
	Post primary	228 (25.3)
Religion	Muslims	382 (42.4)
	Christians	518 (57.6)
Marital status	Single	133 (14.8)
	Married	767 (85.2)
Occupation	Farming	502 (55.8)
	Others (business, housewife, civil servant)	195 (21.7)
Parity	Mean (SD)	5.04 (±2.7)
	Four and below	430 (47.8)
	Above four	470 (52.2)
Household monthly income	Less than $40	622 (69.1)
	$40 and above	278 (30.9)
Household head	No	757 (84.1)
	Yes	143 (15.9)
Ever tested for HIV	No	144 (16.0)
	Yes	756 (84.0)
Ever used modern family planning method	No	317 (35.2)
	Yes	583 (64.8)
Cervical cancer screening status	Not screened	857 (95.2)
	Screened	43 (4.8)

### Intention to screen for cervical cancer

Majority 819 (91.0%) of respondents stated that they intended to go for cervical cancer screening in the following six months. Only 57 (6.3%) did not intend to undergo the procedure while 24 (2.7%) were undecided. Reasons for intention to screen were: 603 (73.6%) to know status, 256 (31.3%) thought it was important, 202 (24.7%) wanted to reduce their chances of getting the disease, and 20 (2.4%) had been told to do so by a health worker. Among those who did not intend to screen, 28 (49.1%) stated that it was not necessary or important, 9 (15.8%) cited health facility being far away, 6 (10.5%) high costs involved, 5 (8.8%) said they had been screened recently, and 13 (22.8%) gave other reasons (fear of pain, lack of signs and symptoms, and already undergoing treatment). Among respondents who had never screened for cervical cancer, intention to screen was at 91.6% (785/857). When respondents were asked whether they would be willing to undergo screening if the service was offered at an affordable cost to them, majority 828 (92.0%) said they would. Moreover, 863 (95.9%) stated willingness to screen if the service was provided free of charge.

### Respondents’ willingness for vaccination of themselves and their daughters

Of the study respondents, more than nine in ten 815 (90.5%) reported willingness to be vaccinated against cervical cancer if it were recommended. The rest reported unwillingness 61 (6.8%) or uncertainty 24 (2.7%). Majority 813 (90.4%) also reported that they would allow their daughters to be vaccinated against cervical cancer, 59 (6.6%) wouldn’t and the rest 28 (3.1%) were undecided.

### Socio-demographic characteristics associated with intention to screen, and willingness to vaccinate daughters against cervical cancer

Respondents from Mayuge district (unadjusted PR = 1.10, 95% CI = 1.06–1.14) and those whose households earned 40 US dollars or more per month (unadjusted PR = 1.07, 95% CI = 1.04–1.11) had a 10% and 7% higher intention to screen for cervical cancer respectively compared with their counterparts. On the other hand, respondents who had accessed cervical cancer screening were less likely to intend to undergo another test in the subsequent six months (unadjusted PR = 0.86, 95% CI = 0.74–0.99). The respondents’ district of residence (unadjusted PR = 1.05, 95% CI = 1.01–1.09), residence in a rural or urban area (unadjusted PR = 1.05, 95% CI = 1.01–1.08), occupation (unadjusted PR = 0.94, 95% CI = 0.91–0.98), and household income (unadjusted PR = 1.05, 95% CI = 1.01–1.08) were all significantly associated with willingness to vaccinate their daughters against cervical cancer (Table 2).

**Table 2:** Knowledge factors with intention to screen and willingness to vaccinate daughters against cervical cancer

	Intention to screen (*n* = 819)			Willingness to vaccinate daughters (*n* = 813)		
Characteristic	N (%)	Unadjusted PR (95% CI)	*p*-value	N (%)	Unadjusted PR (95% CI)	*p*-value
Early detection of cervical cancer is helpful						
No	36 (80.0)			39 (86.7)		
Yes	783 (94.2)	1.18 [1.02–1.36]	0.029*	774 (93.6)	1.08 [0.96–1.21]	0.194
Cervical cancer can be prevented						
No	246 (92.8)			246 (92.1)		
Yes	573 (93.8)	1.01 [0.97–1.05]	0.611	567 (93.7)	1.02 [0.98–1.06]	0.412
Knew recommended age for start of cervical cancer screening						
No	785 (93.6)			782 (93.5)		
Yes	34 (91.9)	0.98 [0.89–1.08]	0.717	31 (86.1)	0.92 [0.81–1.05]	0.221
Knew that one can be vaccinated against cervical cancer						
No	282 (90.7)			280 (90.0)		
Yes	537 (95.0)	1.05 [1.01–1.09]	0.022*	533 (95.0)	1.05 [1.01–1.10]	0.011*
Knew more than one preventive measure for cervical cancer						
No	570 (93.4)			580 (94.5)		
Yes	249 (93.6)	1.00 [0.96–1.04]	0.927	233 (90.3)	0.96 [0.91–0.99]	0.047*
Knew more than one symptom of cervical cancer						
No	382 (95.0)			387 (96.3)		
Yes	437 (92.2)	0.97 [0.94–1.00]	0.085	426 (90.6)	0.94 [0.01–0.97]	0.001*
Knew at least one test for cervical cancer						
No	455 (95.8)			448 (94.9)		
Yes	364 (90.8)	0.95 [0.91–0.98]	0.004*	365 (91.2)	0.96 [0.93–0.99]	0.036*
Knew someone who had ever been screened for cervical cancer						
No	538 (93.6)			546 (94.5)		
Yes	281 (93.4)	0.99 [0.96–1.03]	0.905	267 (90.8)	0.96 [0.92–1.00]	0.062
Knew someone who had ever been diagnosed with cervical cancer						
No	569 (93.3)			576 (93.9)		
Yes	250 (93.9)	1.00 [0.97–1.04]	0.691	237 (91.5)	0.97 [0.93–1.01]	0.218
Knew someone who had died from cervical cancer						
No	568 (93.3)			572 (93.6)		
Yes	251 (94.0)	1.00 [0.97–1.04]	0.676	241 (92.3)	0.99 [0.95–1.03]	0.507

*p < 0.05

### Knowledge factors associated with intention to screen, and willingness to vaccinate daughters against cervical cancer

Reporting that early detection of cervical cancer is helpful (unadjusted PR = 1.18, 95% CI = 1.02–1.36), knowing that one could be vaccinated against cervical cancer (unadjusted PR = 1.05, 95% CI = 1.01–1.09), and knowledge of at least one test for cervical cancer (unadjusted PR = 0.95, 95% CI = 0.91–0.98) were significantly associated with intention to screen for cervical cancer at bivariate analysis. On the other hand, knowing that one could be vaccinated against cervical cancer (unadjusted PR = 1.05, 95% CI = 1.01–1.10), knowing more than one preventive measure (unadjusted PR = 0.96, 95% CI = 0.91–0.99) or symptom of cervical cancer (unadjusted PR = 0.94, 95% CI = 0.01–0.97), and knowledge of at least one test for cervical cancer (unadjusted PR = 0.96, 95% CI = 0.93–0.99) were significantly associated with willingness to vaccinate daughters against cervical cancer (Table 3).

**Table 3:** Attitudes with intention to screen and willingness to vaccinate daughters against cervical cancer

	Intention to screen (*n* = 819)			Willingness to vaccinate daughters (*n* = 813)		
	N (%)	Unadjusted PR (95% CI)	*p*-value	N (%)	Unadjusted PR (95% CI)	*p*-value
Cervical cancer is a severe disease						
No	19 (45.2)			7 (17.1)		
Yes	796 (96.1)	2.12 [1.52–2.96]	<0.001*	800 (96.9)	5.68 [2.89–11.12]	<0.001*
I am at risk of getting cervical cancer						
No	62 (73.8)			47 (56.6)		
Yes	642 (95.8)	1.29 [1.14–1.48]	<0.001*	647 (97.3)	1.72 [1.42–2.07]	<0.001*
Cervical cancer screening is important						
No	20 (52.6)			7 (18.4)		
Yes	788 (95.4)	1.81 [1.34–2.45]	<0.001*	802 (97.3)	5.28 [2.70–10.32]	<0.001*
Only women who are sexually active need cervical cancer screening						
Yes	373 (95.9)			378 (96.9)		
No	358 (90.9)	0.95 [0.91–0.98]	0.005	351 (89.5)	0.92 [0.89–0.96]	<0.001*
Women who have had sexually transmitted infections are more likely to get cervical cancer						
No	169 (86.2)			152 (79.2)		
Yes	578 (96.0)	1.11 [1.05–1.18]	<0.001*	588 (97.7)	1.23 [1.15–1.33]	<0.001*
Chances of curing cervical cancer are better when the disease is discovered at an early stage						
No	97 (78.9)			89 (70.6)		
Yes	666 (96.2)	1.22 [1.11–1.34]	<0.001*	671 (97.7)	1.38 [1.23–1.55]	<0.001*
Cervical cancer is not a death sentence for most people						
No	320 (95.0)			319 (94.9)		
Yes	407 (92.5)	0.97 [0.94–1.01]	0.157	401 (91.3)	0.96 [0.93–0.99]	0.046*
There is much a woman can do to reduce her chances of getting cervical cancer						
No	415 (95.6)			410 (95.1)		
Yes	272 (90.7)	0.95 [0.91–0.99]	0.012*	269 (89.1)	0.94 [0.89–0.98]	0.004*
Women who have cervical cancer will have some symptoms showing it						
Yes	700 (96.1)			706 (97.4)		
No	53 (68.8)	0.71 [0.61–0.83]	<0.001*	41 (54.7)	0.56 [0.46–0.69]	<0.001*
Cervical cancer runs in families						
No	371 (92.7)			361 (91.2)		
Yes	302 (94.4)	1.02 [0.98–1.05]	0.374	307 (95.0)	1.04 [1.00–1.08]	0.039*
Women only need cervical cancer screening tests during child bearing years						
Yes	412 (95.8)			412 (96.5)		
No	321 (89.7)	0.93 [0.89–0.97]	0.001*	318 (89.1)	0.92 [0.89–0.96]	<0.001*
Their family would approve of children being vaccinated against cervical cancer						
No	34 (56.7)			9 (15.5)		
Yes	752 (96.5)	1.70 [1.36–2.13]	<0.001*	778 (98.9)	6.38 [3.49–11.63]	<0.001*

*p < 0.05

### Attitude factors associated with intention to screen and willingness to vaccinate daughters against cervical cancer

Majority 796 (96.1%) of respondents thought that cervical cancer was a severe disease and these had a significantly higher intention to screen (unadjusted PR = 2.12, 95% CI = 1.52–2.96). Other attitudinal factors associated with intention to screen for cervical cancer were: belief of being at risk of getting cervical cancer (unadjusted PR = 1.29, 95% CI = 1.14–1.48), stating that cervical cancer screening is important (unadjusted PR = 1.81, 95% CI = 1.34–2.45), and that chances of curing cervical cancer are better when it is discovered at an early stage (unadjusted PR = 1.22, 95% CI = 1.11–1.34), and that their family would approve of children being vaccinated against cervical cancer (unadjusted PR = 1.70, 95% CI = 1.36–2.13). Similarly, respondents who stated that cervical cancer is a severe disease (unadjusted PR = 5.68, 95% CI = 2.89–11.12), believed were at risk of getting cervical cancer (unadjusted PR = 1.72,, 95% CI = 1.42–2.07), stated that; cervical cancer screening is important (unadjusted PR = 5.28, 95% CI = 2.70–10.32), the chances of curing cervical cancer are better when the disease is discovered at an early stage (unadjusted PR = 1.38, 95% CI = 1.23–1.55), and that their family would approve of children being vaccinated against cervical cancer (unadjusted PR = 6.38, 95% CI = 3.49–11.63) had a significantly higher willingness to vaccinate their daughters against the disease (Table 4).

**Table 4:** Health facility factors with intention to screen and willingness to vaccinate daughters against cervical cancer

	Intention to screen (*n* = 819)			Willingness to vaccinate daughters (*n* = 813)		
Characteristic	N (%)	Unadjusted PR (95% CI)	*p*-value	N (%)	Unadjusted PR (95% CI)	*p*-value
Where health care is accessed when sick						
Private facility	166 (97.1)			168 (96.5)		
Government facility	653 (92.6)	0.95 [0.92–0.99]	0.006*	645 (92.4)	0.96 [0.92–0.99]	0.015*
Where reproductive health care is accessed						
Private facility	153 (96.8)			157 (97.5)		
Government facility	666 (92.8)	0.96 [0.92–0.99]	0.016*	656 (92.3)	0.95 [0.91–0.98]	0.001*
Ease of access of reproductive health care						
Very/somewhat difficult	372 (92.8)			378 (94.9)		
Not difficult	447 (94.1)	1.01 [0.98–1.05]	0.428	435 (91.8)	0.97 [0.93–1.00]	0.056
Have challenges in accessing reproductive health care						
Yes	480 (92.5)			487 (94.0)		
No	339 (95.0)	1.02 [0.99–1.06]	0.131	326 (92.1)	0.98 [0.94–1.02]	0.279
Ever been recommended for screening by health worker						
No	749 (94.0)			737 (92.9)		
Yes	70 (88.6)	0.94 [0.87–1.02]	0.155	76 (96.2)	1.03 [0.99–1.08]	0.157
Knew where cervical cancer screening is provided						
No	496 (95.7)			489 (95.9)		
Yes	323 (90.2)	0.94 [0.91–0.98]	0.003*	324 (89.5)	0.93 [0.89–0.97]	0.001*
Distance to health facility where screening is done (*n*–372)						
5 or more km	140 (84.8)			139 (84.2)		
Less than 5 km	183 (94.8)	1.12 [1.04–1.20]	0.003*	187 (94.9)	1.13 [1.05–1.21]	0.001*
Distance to nearest health facility						
5 or more km	128 (91.4)			125 (89.9)		
Less than 5 km	691 (93.9)	1.03 [0.97–1.08]	0.336	688 (93.9)	1.04 [0.98–1.10]	0.153

*p < 0.05

### Health facility factors associated with intention to screen and willingness to vaccinate against cervical cancer

Respondents who accessed health care (unadjusted PR = 0.95, 95% CI = 0.92-0.99) and reproductive health care (unadjusted PR = 0.96, 95% CI = 0.92–0.99) from a government health facility, and those who knew where cervical cancer screening was provided (unadjusted PR = 0.94, 95% CI = 0.91–0.98) had a lower intention to screen for cervical cancer compared with their counterparts. On the other hand, respondents who lived within 5 kilometres from the health facility where screening services were provided had a higher intention to screen for cervical cancer (unadjusted PR = 1.12, 95% CI = 1.04–1.20). Similarly, respondents who lived within 5 kilometres from the health facility had a higher willingness for vaccination of their daughters (unadjusted PR = 1.13, 95% CI = 1.05–1.21). In contrast, there was less willingness for respondents who accessed health care (unadjusted PR = 0.96 95% CI = 0.92–0.99) and reproductive health care (unadjusted PR = 0.95, 95% CI = 0.91-0.98) from a government health facility, those who found it easy to access reproductive health care (unadjusted PR = 0.97, 95% CI = 0.93–1.00), and those who knew where cervical cancer screening was provided (unadjusted PR = 0.93, 95% CI = 0.89–0.97) (Table 5).

**Table 5:** Socio-demographic characteristics with intention to screen, and willingness to vaccinate daughters against cervical cancer

Characteristic	Intention to screen (*n* = 819)			Willingness to vaccinate daughters (*n* = 813)		
	N (%)	Unadjusted PR (95% CI)	*p*-value	N (%)	Unadjusted PR (95% CI)	*p*-value
District						
Bugiri	388 (89.0)			404 (90.9)		
Mayuge	431 (97.9)	1.10 [1.06–1.14]	<0.001*	409 (95.6)	1.05 [1.01–1.09]	0.007*
Residence						
Rural	551 (92.6)			542 (91.9)		
Semi-urban/urban	268 (95.4)	1.03 [0.99–1.06]	0.093	271 (96.1)	1.05 [1.01–1.08]	0.009*
Age (years)						
25–39	643 (93.7)			638 (93.5)		
40–49	176 (92.6)	0.99 [0.94–1.03]	0.603	175 (92.1)	0.98 [0.94–1.03]	0.508
Education level						
None/primary	617 (94.0)			610 (93.7)		
Post primary	202 (91.8)	0.98 [0.93–1.02]	0.283	203 (91.9)	0.98 [0.94–1.02]	0.376
Religion						
Muslims	353 (94.9)			350 (94.6)		
Christians	466 (92.5)	0.97 [0.94–1.00]	0.138	463 (92.2)	0.97 [0.94–1.01]	0.159
Marital status						
Single	118 (92.9)			120 (93.7)		
Married	701 (93.6)	1.01 [0.96–1.06]	0.782	693 (93.1)	0.99 [0.95–1.04]	0.795
Occupation						
Farming	458 (94.2)			470 (95.5)		
Other (business, housewife, civil servant)	361 (92.6)	0.98 [0.95–1.02]	0.325	343 (90.3)	0.94 [0.91–0.98]	0.004*
Parity						
Above four	426 (92.4)			429 (93.5)		
Four and below	393 (94.7)	1.02 [0.99–1.06]	0.167	384 (92.9)	0.99 [0.96–1.03]	0.776
Household monthly income						
Less than $40	551 (91.4)			552 (91.8)		
$40 and above	268 (98.2)	1.07 [1.04–1.11]	<0.001*	261 (96.3)	1.05 [1.01–1.08]	0.005
Household head						
No	688 (93.3)			688 (93.3)		
Yes	131 (94.2)	1.01 [0.96–1.06]	0.681	125 (92.6)	0.99 [0.94–1.04]	0.756
Ever tested for HIV						
No	133 (94.3)			133 (95.0)		
Yes	686 (93.3)	0.99 [0.95–1.03]	0.644	680 (92.9)	0.98 [0.94–1.02]	0.307
Ever used modern family planning method						
No	283 (92.5)			279 (91.8)		
Yes	536 (94.0)	1.02 [0.98–1.06]	0.392	534 (94.0)	1.02 [0.98–1.06]	0.233
Cervical cancer screening status						
Not screened	785 (94.1)			774 (93.2)		
Screened	34 (80.9)	0.86 [0.74–0.99]	0.046*	39 (92.9)	0.99 [0.91–1.08]	0.923

*p < 0.05

### Predictors of intention to screen and willingness to vaccinate daughters against cervical cancer

When all factors were examined simultaneously in relation to intention to screen, respondents whose households earned 40 US dollars and above per month had a significantly higher intention to screen for cervical cancer compared to those who earned less than 40 dollars (adjusted PR = 1.11, 95% CI: 1.03–1.20). Cervical cancer screening status (adjusted PR = 0.81, 95% CI: 0.67–0.99) and knowledge of at least one test for cervical cancer was significantly associated with intention to screen (adjusted PR = 0.92, 95% CI: 0.85–0.98). Among attitude factors, although not statistically significant, respondents who stated that cervical cancer was a severe disease had an 81% higher intention to screen (adjusted PR = 1.81 95% CI: 0.96–3.42) while the prevalence of intention to screen was 37% higher among respondents whose families would approve of children being vaccinated against cervical cancer (adjusted PR = 1.37, 95% CI: 0.83–2.26) compared with their counterparts.

No sociodemographic characteristic was associated with willingness to vaccinate daughters against cervical cancer. Additionally, respondents who knew more than one symptom of cervical cancer had a lower prevalence of willingness to vaccinate daughters against cervical cancer (adjusted PR = 0.89, 95% CI: 0.81–0.98). Although not statistically significant, the prevalence of willingness to vaccinate daughters was over 4 times higher among respondents who stated that cervical cancer screening was important (adjusted PR = 4.36, 95% CI: 0.38–49.2) and over 3 times higher among those whose families would approve of children being vaccinated against cervical cancer (adjusted PR = 3.87, 95% CI: 0.79–19.04) compared with their counterparts. No attitude and health facility factors were associated with willingness to vaccinate daughters against cervical cancer (Table 6).

**Table 6:** Independent predictors of intention to screen and willingness to vaccinate daughters against cervical cancer

	Intention to screen		Willingness to vaccinate	
Characteristic	Adjusted PR (95% CI)	*p*-value	Adjusted PR (95% CI)	*p*-value
District (Bugiri)				
Mayuge	1.06 [0.99–1.13]	0.072	1.05 [0.97–1.13]	0.248
Residence (Rural)				
Semi-urban/urban	1.05 [0.98–1.14]	0.155	-	-
Age in years (25–39)				
40–49	1.01 [0.92–1.12]	0.845	1.04 [0.99–1.10]	0.133
Education level (None/primary)				
Post primary	0.93 [0.84–1.03]	0.170	0.97 [0.92–1.02]	0.231
Religion (Muslims)				
Christians	0.92 [0.85–1.00]	0.059	0.97 [0.93–1.01]	0.172
Household monthly income (Less than $40)				
$40 and above	1.11 [1.03–1.20]	0.004*	1.03 [0.99–1.07]	0.149
Cervical cancer screening status (No)				
Yes	0.81 [0.67–0.99]	0.037*	1.01 [0.93–1.11]	0.769
Knowledge factors				
Early detection of cervical cancer is helpful (No)				
Yes	1.07 [0.92–1.24]	0.407	-	-
Knew that one can be vaccinated against cervical cancer (No)				
Yes	1.00 [0.92–1.08]	0.998	0.99 [0.96–1.04]	0.959
Knew more than 1 preventive measure for cervical cancer (No)				
Yes	-	-	1.04 [0.99–1.09]	0.136
Knew more than 1 symptom of cervical cancer (No)				
Yes	0.93 [0.86–1.01]	0.078	0.89 [0.81–0.98]	0.020*
Knew at least one test for cervical cancer (No)				
Yes	0.92 [0.85–0.98]	0.017*	0.99 [0.93–1.05]	0.655
Attitude factors				
Cervical cancer is a severe disease (No)				
Yes	1.81 [0.96–3.42]	0.068	1.36 [0.79–2.30]	0.258
I am at risk of getting cervical cancer (No)				
Yes	0.89 [0.78–1.03]	0.127	1.04 [0.86–1.27]	0.660
Cervical cancer screening is important (No)				
Yes	0.61 [0.34–1.09]	0.097	4.36 [0.38–49.2]	0.234
Only women who are sexually active need cervical cancer screening (Yes)				
No	-	-	1.02 [0.97–1.06]	0.479
Women who have had sexually transmitted diseases are more likely to get cervical cancer (No)				
Yes	1.05 [0.97–1.14]	0.190	1.03 [0.97–1.09]	0.303
Chances of curing cervical cancer are better when the disease is discovered at an early stage (No)				
Yes	0.99 [0.92–1.08]	0.947	0.97 [0.91–1.03]	0.338
Cervical cancer is not a death sentence for most people (No)				
Yes	-	-	0.99 [0.95–1.04]	0.790
There is much a woman can do to reduce her chances of getting cervical cancer (No)				
Yes	1.02 [0.95–1.08]	0.697	1.00 [0.97–1.03]	0.929
Women who have cervical cancer will have some symptoms showing it (Yes)				
No	0.86 [0.71–1.06]	0.157	0.86 [0.72–1.02]	0.085
Cervical cancer runs in families (No)				
Yes	-	-	0.98 [0.95–1.02]	0.410
Women only need cervical cancer screening tests during child bearing years (No)				
No	0.99 [0.92–1.06]	0.802	0.97 [0.93–1.02]	0.228
Their family would approve of children being vaccinated against cervical cancer (No)				
Yes	1.37 [0.83–2.26]	0.217	3.87 [0.79–19.04]	0.096
Health facility factors				
Where health care is accessed (Private facility)				
Government facility	0.93 [0.82–1.05]	0.240	1.03 [0.87–1.20]	0.749
Where reproductive health care is accessed (Private facility)				
Government facility	1.11 [0.89–1.39]	0.351	0.95 [0.86–1.05]	0.347
Knew where cervical cancer screening is provided (No)				
Yes	1.13 [0.97–1.32]	0.121	1.08 [0.96–1.23]	0.189
Distance to health facility where screening is done (5 or more km)				
Less than 5 km	1.07 [0.98–1.17]	0.124	1.03 [0.98–1.08]	0.271

*p < 0.05

## Discussion

This study assessed the socio-demographic, knowledge, attitudinal and health facility factors associated with women’s intention to screen, and willingness to vaccinate their daughters against cervical cancer in eastern Uganda. Overall, 91% of respondents reported intention to screen for cervical cancer in the subsequent six months. This intention to screen increased to 92% if the service would be offered at an affordable cost, and to 95.9% if provided free of charge. This intention to screen for cervical cancer is higher than the 63% reported by a previous study conducted in southern Ugandan [18]. Although intention to screen was this high and respondents relatively knowledgeable about cervical cancer and screening [35], actual uptake of cervical cancer screening in this population of women was very low at 4.8% as reported in a related study [19]. This therefore calls for an in-depth exploration of the barriers that could be limiting most of these women from accessing cervical cancer screening. In fact, respondents who did not intend to screen majorly stated that screening was neither necessary nor important highlighting the presence of knowledge gaps that should be filled through adequate sensitisation of women to appreciate the severity of the disease and increase their risk perception. Therefore, increasing access to cervical cancer screening services and further education of women is expected to increase uptake rates of screening services which are currently low in developing countries including Uganda.

The target group for HPV vaccinations being prepubescent girls, parental approval is required for the success of vaccination programmes [24]. In this study, we found a high willingness of 90.4% among women to vaccinate their daughters against cervical cancer. This is in line with proportions of over 90% reported by Kenyan [36, 37] and Ghanaian [7] studies. However, these studies majorly targeted urban women at health centres unlike our study that had majority rural participants in the community. This high willingness in our study could have been contributed to by the high acceptability and uptake of vaccines in Uganda [5], a determinant of HPV vaccine acceptability among parents [7, 25, 28]. There are also ongoing campaigns by the government of Uganda aimed at HPV vaccinations of girls aged between 9 to 13 years which could have increased awareness of cervical cancer and vaccinations. As there is high willingness to vaccinate daughters, the focus of various stakeholders such as the Ministry of Health and Non-governmental Organisations should be turning this into actual practice through increasing service availability and accessibility by using effective strategies such as schools, health centres, and community based programmes [5, 8].

In the study, there was a significant association between having higher income and intention to screen against cervical cancer. Moreover, intention to screen increased with possibilities of affordable and free cervical cancer screening which could highlight the cost of the procedure as a barrier as reported elsewhere [16, 19, 38]. Prior studies have also found associations between higher income and uptake of cervical cancer screening [17, 39, 40]. This highlights the need to ensure the provision of affordable or free cervical cancer screening services to increase uptake of the service among women in developing countries. We also found that respondents who had already accessed screening, and those who knew at least one test for cervical cancer were less likely to intend to screen in the subsequent six months. It is likely that respondents who knew the screening tests had been screened for cervical cancer as earlier highlighted [19]. Since these respondents had already been screened, though at various intervals [19], they could have been reluctant to access screening in the following six months. Indeed, the WHO recommends screening for cervical cancer at 3 year intervals among women aged 30 years and above [11]. Similarly, the Ugandan Ministry of Health strategic plan for cervical cancer prevention and control 2010–2014 recommends screening for cervical cancer once every three years for HIV negative women, and annually for those who are HIV positive [6]. Nonetheless, a study in Kenya [17] found lower intentions to screen among women who had already been screened while a Chinese study reported otherwise [41]. Respondents who knew more than one symptom of the disease had lower willingness to vaccinate their daughters. It is possible that such respondents were reluctant to take up interventions in cases where they knew how the disease could manifest yet they had not observed these. Indeed, it has been reported that women may for example not access cervical cancer screening because they do not have any signs and symptoms of the disease [18, 19]. Sensitization campaigns should emphasize the asymptomatic nature of cervical cancer especially during its early stages and encourage women to access cervical cancer services even when they do not have any signs or symptoms of the disease. Among the attitudinal factors, although not statistically significant, respondents whose families would approve of daughters being vaccinated against cervical cancer were more likely to intend to screen for the disease, and more willing to have their daughters vaccinated against it. Study respondents being women, this could indicate a gender influence which has been shown to affect health care decision making in many African households [42, 43]. For example, previous studies have shown that sometimes women may require approval and financial support from their husbands to seek cervical cancer screening [18, 42, 43] emphasizing the importance of social influence from important others such as spouses. On the other hand, this finding could indicate the desire for collective decision making among parents especially when it comes to the vaccination of their daughters as reported by another study [7]. Overall, this underscores the need for involvement of all societal groups especially men in programmes aimed at increasing cervical cancer screening and vaccination.

This study examined intention to screen, and willingness to vaccinate daughters among a majorly rural population, and had a large sample size compared with other sub-Saharan Africa studies. However, some elements of this study for example intention to screen, and willingness to vaccinate daughters could have been affected by the tendency for study participants to give socially desirable responses which could have introduced bias. We also did not assess knowledge about HPV and the vaccine whose relationship with vaccination acceptability would have been relevant to explore, and neither did we assess reasons for willingness to vaccinate daughters among the sampled women. Nevertheless, this study contributes important information regarding intention to screen among women and willingness to vaccinate daughters against cervical cancer and associated factors which can inform future studies as well as cervical cancer programmes.

## Conclusion

There was a very high intention to screen and willingness to vaccinate daughters against cervical cancer among women in eastern Uganda. Only higher income predicted intention to screen for cervical cancer while respondents whose families would approve of their daughters being vaccinated against cervical cancer were more likely to intend to screen for the disease and willing for their daughters to be vaccinated. To take advantage of the high rates of intention to screen, and willingness to vaccinate daughters against cervical cancer, opportunities should be availed for women to access screening, and for girls to be vaccinated against the disease. In addition, the involvement of all societal groups especially men in programmes aimed at increasing screening and vaccinations of girls could increase uptake of these services.

## Abbreviations

HIV: Human immunodeficiency virus
HPV: Human papilloma virus
PR: Prevalence ratio
WHO: World Health Organization
